# Thrombospondin-2 holds prognostic value and is associated with metastasis and the mismatch repair process in gastric cancer

**DOI:** 10.1186/s12885-022-09201-3

**Published:** 2022-03-07

**Authors:** Xiao-dong Chu, Zheng-bin Lin, Ting Huang, Hui Ding, Yi-ran Zhang, Zhan Zhao, Shu-chen Huangfu, Sheng-hui Qiu, Yan-guan Guo, Xiao-li Chu, Jing-hua Pan, Yun-long Pan

**Affiliations:** 1 Department of General Surgery, The First Affiliated Hospital of Jinan University, 613 Huangpu West Avenue, Guangzhou, Guangdong 510632 China; 2 Department of Clinical Pathology, The First Affiliated Hospital of Jinan University, 613 Huangpu West Avenue, Guangzhou, Guangdong 510632 China; 3 Guangdong Provincial Key laboratory of Chinese Medicine for Prevention and Treatment of Refractory Chronic Diseases, The Second Affiliated Hospital of Guangzhou University of Chinese Medicine, Guangzhou, 510120 China

**Keywords:** Gastric cancer, Thrombospondin 2, Clinical characteristics, Prognosis, Lymphatic metastasis

## Abstract

**Background:**

This study aims to investigate thrombospondin 2 (TSP2) expression levels in gastric cancer (GC) and determine the relationship between TSP2 and clinical characteristics and prognosis.

**Methods:**

The online database Gene Expression Profile Interactive Analysis (GEPIA) was used to analyse TSP2 mRNA expression levels in GC. The Kaplan–Meier plotter prognostic analysis tool was used to evaluate the influence of TSP2 expression on clinical prognosis in GC patients. TSP2 expression levels were analysed in paraffin-embedded GC samples and adjacent normal tissues by immunohistochemistry. The relationship between the clinicopathological characteristics and prognosis of GC patients was assessed. Transwell experiments were used to evaluate the effect of TSP2 on HGC27 and AGS cell invasion and migration. The EdU experiment was used to detect the effect of transfection of TSP2 on cell proliferation, and the flow cytometry experiment was used to detect the effect of TSP2 on cell apoptosis and the cell growth cycle. Western blotting (Wb) technology was used to detect MMP, E-cadherin, N-cadherin, Vimentin, Snail, AKT, PI3K, and VEGF protein expression in HGC27 cells.

**Results:**

Compared with normal tissues, TSP2 mRNA expression in GC was significantly upregulated and was closely related to the clinical stage of GC. High TSP2 expression significantly affected the OS, FP and PPS of patients with GC. Among these patients, TSP2 expression levels did not affect the prognosis of patients with GC in the N0 subgroup but significantly affected the prognosis of patients with GC in the N (1 + 2 + 3) subgroup. TSP2 protein expression levels were significantly higher in GC tissue compared with normal tissues (*P <* 0.01). The overall survival (OS) and relapse-free survival (RFS) of patients with high TSP2 expression were lower than those of patients with low TSP2 expression. Cells transfected with the TSP2-silencing sequence exhibited increased apoptosis and inhibition of proliferation, migration and invasion. AKT and PI3K expression in cells was significantly downregulated (*P <* 0.01). AKT, PI3K and VEGF expression in cells transfected with the TSP2 silencing sequence was significantly reduced. Proliferation, migration, invasion ability, and TSP2 expression levels significantly correlated with mismatch repair genes, such as *PMS2, MSH6, MSH2,* and *MLH1* (*P <* 0.05).

**Conclusion:**

TSP2 expression is significantly increased in GC. TSP2 expression is closely related to metastasis and the mismatch repair process in GC patients and affects GC patient prognosis. The mechanism may involve regulating gastric cancer cell proliferation and migration by modulating the VEGF/PI3K/AKT signalling pathway. TSP2 is a potential marker and therapeutic target for the prognosis of GC patients.

**Supplementary Information:**

The online version contains supplementary material available at 10.1186/s12885-022-09201-3.

## Background

Gastric cancer (GC) is one of the most common cancers in the world. With greater than 1 million estimated new cases annually, GC is the fifth most diagnosed malignancy worldwide [1]. Although the development of surgical techniques and combined chemotherapy has made significant progress in the treatment of GC in recent years, the prognosis of patients with advanced GC remains abysmal [2]. At present, GC-specific treatment targets and precise prognostic markers are lacking. GC remains the third leading cause of cancer-related mortality worldwide with a high mortality rate mostly due to its detection in advanced stages of the disease [3]. Therefore, exploring new prognostic biomarkers and developing therapeutic targets are of great significance for the diagnosis and treatment of GC.

Tumour progression involves a series of complex events, starting with tumour cell mutations and ending with invasion and metastasis to distant locations. In this process, the normal tissue structure is destroyed, and the surrounding tissues begin to produce a proliferative response similar to wound healing. This response can be triggered by the highly permeable blood vessels that characterize the tumour vasculature [4]. Tumours are thought to secrete many angiogenic factors. Angiogenesis is regulated by the balance of a variety of proangiogenic factors and inhibitors. These blood vessels release plasma protein, which initiates the production of fibrin [5]. The tumour microenvironment also includes activated immune cells, fibroblasts, extracellular matrix, and newly formed capillaries, which constitute the proliferation response of connective tissue [6]. Although the basis of tumorigenesis and development has been clarified in many aspects, the molecular genetic basis of tumorigenesis and development is still not completely clear. It is widely accepted that cancer is caused by different mutations in specific genes. There is no doubt that the genetic basis of cancer is an important cause of cancer because it results in numerous molecular changes inherent in basic cellular processes [7].

Studies have shown that thrombospondin-2 (TSP2) may be closely related to tumour occurrence and development [8]. TSP2 is one of the five members of the human TSP protein family, namely, TSP1 (THBS1) and TSP2 (THBS2), TSP3, TSP4, and TSP5 [9]. Thrombospondin (TSP) is a stromal cell protein. Its spatial structure is relatively stable, and TSP participates in the communication between cells and the intercellular matrix. Its main functions involve early embryonic development, damage repair, and tumorigenesis [10, 11]. The molecular weight of TSP2 is approximately 145 kD; it is a trimeric structure sensitive to Ca2+ and maintained by disulphide bonds. TSP2 has four protein binding domains similar to TSP1, including the N-terminal heparin-binding domain and lysin-like domain, epidermal growth factor-like domain, and the Ca2+ binding domain [12]. These domains regulate various biological functions, including proliferation, angiogenesis, cell adhesion, and extracellular matrix remodelling, based on interactions with various cell surface receptors. For example, TSP2 interacts with the cytokines CD47, CD36, and integrin αvβ3 to promote cell migration [13]. Studies have shown that the TSP2 gene is closely related to the occurrence and development of coronary atherosclerosis, liver disease, and chronic kidney disease [14]. Further studies have found that TSP2 mRNA expression is abnormally increased in prostate cancer [15] and oral cancer [16] tissues and affects the prognosis of patients, indicating that TSP2 may be closely related to the occurrence and development of tumours, as mentioned above. However, few reports about TSP2 in GC are limited. The relationship between GC is still worthy of further discussion. Therefore, we sought to reveal the clinical significance of TSP2 and its role in GC.

The DNA mismatch repair (MMR) system is necessary to maintain genome stability. Broadly speaking, all the main functions of the MMR system, including correcting biosynthetic errors, monitoring DNA damage, and preventing recombination between different sequences, serve this important purpose. Failure to complete these functions may lead to cancer [17, 18]. Microsatellite instability is associated with 10 to 15% of cases of colorectal cancer, endometrial cancer, ovarian cancer, and gastric cancer. Because the postreplication mismatch repair (MMR) system is defective and cannot be corrected, mutations in microsatellites related to key target genes are thought to have a pathogenic role in the evolution of MMR-deficient tumours [19, 20]. However, the relationship between TSP2 and MMR system in patients with gastric cancer has not been reported yet.

To verify the above hypothesis, this study used bioinformatics technology combined with clinical data to preliminarily analyse TSP2 expression in GC tissues and explore the possible relationship between TSP2 expression and the clinicopathological characteristics and clinical prognosis of GC patients. In addition, HGC-27 and AGS GC cell lines were used to inhibit the potential target TSP2 and observe the in vitro effects of TSP2 on GC cells. This study provides clues and ideas for further study of the mechanism of the TSP2 gene in GC.

## Methods

### Tumour database

The online database Gene Expression Profile Interactive Analysis (GEPIA, http://gepia.cancer-pku.cn/index.html) was used to analyse and compare TSP2 expression levels in GC and normal gastric tissues [21]. The Kaplan–Meier Plotter prognostic analysis tool (http://kmplot.com/analysis/) was used to evaluate the effect of TSP2 expression on the prognosis of GC patients [22]. The Kaplan–Meier Plotter database was also used to analyse the correlation between the TSP2 expression level and clinical characteristics of GC patients.

### Clinical data and follow-up

Using a random number table, 80 GC patients who underwent surgery in the General Surgery Department of the First Affiliated Hospital of Jinan University from January 2016 to December 2017 without prior chemotherapy or radiotherapy were selected. Eighty samples of GC tumour tissue and paired adjacent tissues (3 cm from the edge of the cancerous tissue) were collected. Eighty GC tissues and paired adjacent tissues were fixed with formalin and embedded in paraffin. The pathology department of our hospital confirmed these diagnoses. The staging was unified according to the eighth edition TNM staging standard of the International Union Against Cancer (UICC), and postoperative adjuvant treatment was performed according to the National Comprehensive Cancer Network (NCCN) GC practice guidelines. The endpoint of this study was the follow-up period of four years or the patient’s death. Overall survival (OS) was defined as the period from the day of surgery until death from any cause or the end of the follow-up. The Institutional Review Board approved this study of the First Affiliated Hospital of Jinan University, and all of the patients provided informed consent.

### Immunohistochemical test

Take paraffin sections of GC tissue, make 4 μm-thick paraffin sections, and bake the slices at 65 °C for 30 min. After dewaxing, block the endogenous peroxidase with 3% H2O2, inactivate for 10 min, and rinse twice with PBS. Slices are placed 0.01 mol/L (pH 6.0) citrate buffer 90 °C–95 °C heating for 15 min to perform antigen retrieval. Wash twice with PBS. Nonspecific antigens were blocked with 5% BSA. Samples were incubated in rabbit anti-human TSP2 monoclonal antibody (BW1441, Santa Cruz) diluted 1:400 with 5% BSA. The tissue was completely covered in this solution. Samples were incubated overnight in a refrigerator at 4 °C and rinsed twice with PBS (5 min). The secondary antibody (goat anti-rabbit) was added to the glass slide to completely cover the tissue. Samples were incubated at 37 °C for 40 min and rinsed with PBS twice (5 min). DAB was used to develop colour, and the cells were observed under a microscope. The reaction time was 2–4 min, and the reaction was stopped by washing with tap water. The sample was counterstained with haematoxylin at room temperature, washed with tap water, dehydrated with gradient ethanol solution, cleared with xylene, mounted with neutral gum, and observed under a microscope. The immunohistochemical staining area was scored as follows according to the percentage of positive cells: 0 (0%), 1 (1–25%), 2 (26–50%), 3 (51–75%) and 4 (76–100%). The TSP2 staining intensity score was 0 (no staining), 1 (weak staining), 2 (medium staining), and 3 (strong staining). The final staining score is the product of two parameters divided into 2 groups: groups with a total score of 0–3 are low expression groups, and groups with a score of ≥4 are high expression groups.

### Cell culture and transfection

Human HGC-27 and AGS GC cell lines were purchased from ATCC, and grown in 1640 medium supplemented with 10% foetal bovine serum and 1% penicillin-streptomycin mixture (purchased from Guangzhou Genio Biotech Co., Ltd.). All cells were cultured in a 37 °C, 5% CO2 constant temperature incubator, and the medium was changed every 2 days. Cells were observed under a microscope. After the cells reached 80–90% confluence, 0.25% trypsin was used (purchased from Guangzhou Genio Biotech Co., Ltd.) to digest and continue subculture at a ratio of 1:2. Cell transfection: Human HGC27 and AGS GC cells were cultured in 1640 medium containing 10% foetal bovine serum to a confluency of approximately 70%. Then, the target siRNA was used according to the manufacturer’s instructions (purchased from Guangzhou Genio Biotechnology Co., Ltd.). TSP2 siRNA was transfected into GC cells using Lipo3000 liposomes. The specific interference sequence and control sequence were as follows: Si-1 (5′-CCGGCCCTCCTAAGACAAGGAACATCT-3′); Si-2 (5′-CGAGATGTTCCTTGTCTTAGGAGGGTTTTTG-3′); and control group (Ctrl) (5′-CCCTCCTAAGACAAGGAACAT-3′). The obtained stably transfected cells were named Si-1 and Si-2, respectively.

### Western blotting

After 48 h of cell transfection, the total protein was extracted from protein lysate. The sample and loading buffer were mixed according to the corresponding ratio, and then the sample was denatured in a boiling water bath. Each lane was loaded with an equal amount of protein (30 μg). After electrophoresis, the proteins were transferred to PVDF membranes. After blocking with 5% skimmed milk powder, blots were incubated in the corresponding primary antibody (1:1000, ab112543, Abcam) followed by the secondary antibody the next day. Then, ECL developer solution was added in a dark environment, and the blot was exposed using a gel imager. The final result is expressed as a target strip. The ratio of the optical density of the target band to that of the internal control GAPDH (1:2000, AF1186, Biyuntian) was reported as the protein expression level.

### Cell migration and invasion experiments

After transfection for 48 h, HGC-27 and AGS cell lines were digested with 0.25% trypsin. Then, the digestion was terminated, and the culture medium was discarded by centrifugation. The cells were resuspended in serum-free 1640 medium, and the cell density was adjusted to 1 × 10^6^/ml. After repeated pipetting and mixing of the cell suspension, 0.2 ml of the cell suspension was added to the upper chamber of the Transwell chamber. Then, 0.6 ml of 1640 medium containing 10% serum was added to the lower chamber of the 24-well plate. Then, the plate was gently shaken and placed in an incubator for 24 h. The Transwell chamber was removed, and the culture medium in the well was discarded. The cells in the upper chamber were gently removed with a cotton swab. Cells were rinsed 3 times with PBS and fixed with 4% paraformaldehyde for 25 min. Then, the chamber is properly dried, and the cells are stain with 0.1% crystal violet for 20 min. Cells are washed thrice in PBS, and the chamber is air dried. The chamber is placed under a microscope. Five fields of view are randomly selected to observe the cells, take pictures, and count them. In the invasion experiment, Matrigel was added to the Transwell chamber, and the treatment method was the same as described above. Then, 0.2 ml of cell suspension was added to the upper chamber, and the remaining methods were the same as described above.

### EdU assay

A total of 5000 HGC-27 cells were plated in each well of 6-well plates and treated with ethanol (50 μM, 48 h), transfected with Ctrl (100 nM, 48 h), or transfected with si-1 or si-2 (100 nM, 48 h). An EdU staining proliferation kit was purchased from Abcam (ab222421). EdU solution was added to the plates. Plates were incubated for 3 h and then treated with 4% formaldehyde. After the process, the cells were stained with DAPI and viewed under an inverted microscope (Olympus, Japan). Each experiment was repeated three times.

### Flow cytometry

After transfection for 48 h, the cells were collected for single-cell suspension and centrifuged at 1500 r min − 1 for 5 min after rinsing three times with cold PBS. The supernatant was discarded, and the cells were resuspended in PBS at a density of 1 × 106/mL. The cells were fixed by adding − 20 °C precooled 75% ethanol at 4 °C for 1 h followed by centrifugation. After rinsing twice with PBS, the supernatant was discarded, and the cells were incubated with 100 μL RNase in darkness followed by a 30-min water bath. Subsequently, 400 μL propidium iodide (PI) (Sigma, 5 mg/100 mL) was added, and the mixture was incubated in darkness at 4 °C for 30 min for detection. The cells (1 × 10^4^) were evaluated using flow cytometry (6HT, Wuhan Cellwar Biotechnology Co., Ltd., Wuhan, China) with a 350 mesh sieve. Fluorescent signal intensity at an excitation wavelength of 488 nm was recorded to evaluate the cell cycle.

After transfection for 48 h, cells were digested with trypsin without ethylenediaminetetraacetic acid (EDTA), collected in a flow tube, and centrifuged at 2000 rnmin− 1 for 8 min at room temperature. After being washed, the cells were resuspended in precooled PBS (4 °C) and centrifuged at 2000 r min − 1 for 5 min, and the supernatant was discarded. The cells were collected and stained according to the Annexin-V-FITC cell apoptosis detection kit (Sigma) with Annexin-V-FITC/PI staining solution containing Annexin-V-FITC, PI, and HEPES at a ratio of 1:2:50. Briefly, 100 μL staining solution was used to resuspend 1 × 106 cells. Once the solution was completely mixed and incubated at room temperature for 15 min, 1 mL HEPES buffer solution was added to the cells and mixed. Flow cytometry was used to evaluate cell apoptosis with an excitation wavelength of 488 nm. The procedure was repeated thrice.

### Real-time quantitative polymerase chain reaction (PCR)

After that, the cells were collected, and RNA was extracted using the TRIzol method. After the purity and integrity of the RNA had been determined, cDNA was prepared through reverse transcription. The PCR conditions were the following: predenaturation at 95 °C for 1 min; denaturation at 95 °Cfor 15 s, annealing at 58 °C for 20 s, extension at 72 °C for 20 s, for 40 cycles; then, extension at 72 °C for 5 min to terminate the reaction. After the completion of real-time quantitative PCR (RT-qPCR), the reliability of the melting curve and amplification curve results obtained by PCR was quantitatively analyzed, and the cycle threshold (Ct) was set. There were three duplicate holes in each group, and the test was repeated 3 times. The sequence details:TSP2(Forward: 5′-GGG GAC ACT TTG GAC CTC AAC-3′;Reverse: 5′-GCA GCC CAC ATA CAG GCT A-3′);GAPDH (Forward: 5′-ACA ACT TTG GTA TCG TGG AAGG-3′;Reverse: 5′-GCC ATC ACG CCA CAG TTT C-3′).

### Statistical analysis

SPSS 22.0 (IBM Corp., Armonk, NY, USA) and GraphPad Prism 7 (GraphPad Software, Inc., San Diego, CA, USA) were used for data analysis and graphing. The differences between groups were assessed using a t-test or one-way analysis of variance. The expression level of related genes and the characteristic clinicopathological parameters were compared using Fisher’s exact test or χ2 test. Kaplan–Meier survival curves were used to analyse the relationship between TSP2 expression level and OS. *P <* 0.05 indicates a statistically significant difference.

## Results

### Analysis of TSP2 expression levels in different tumours

GEPIA database analysis of TSP2 expression level in tumours showed that TSP2 gene expression was significantly increased in a variety of tumours (Supplementary Fig. 1A, C), and TSP2 expression was significantly higher in GC (stomach adenocarcinoma; STAD) samples compared with normal tissues (*P <* 0.01) (Supplementary Fig. 1B, C). Further comparison of the TSP2 expression levels of different GC clinical stages revealed showed that TSP2 expression was statistically significant in different stages (F = 3.16, *P* = 0.0248, Supplementary Fig. 1D). TSP2 expression in GC tissue from II-IV stage disease was significantly increased.

### The relationship between TSP2 expression level and GC prognosis

**Fig. 1:**
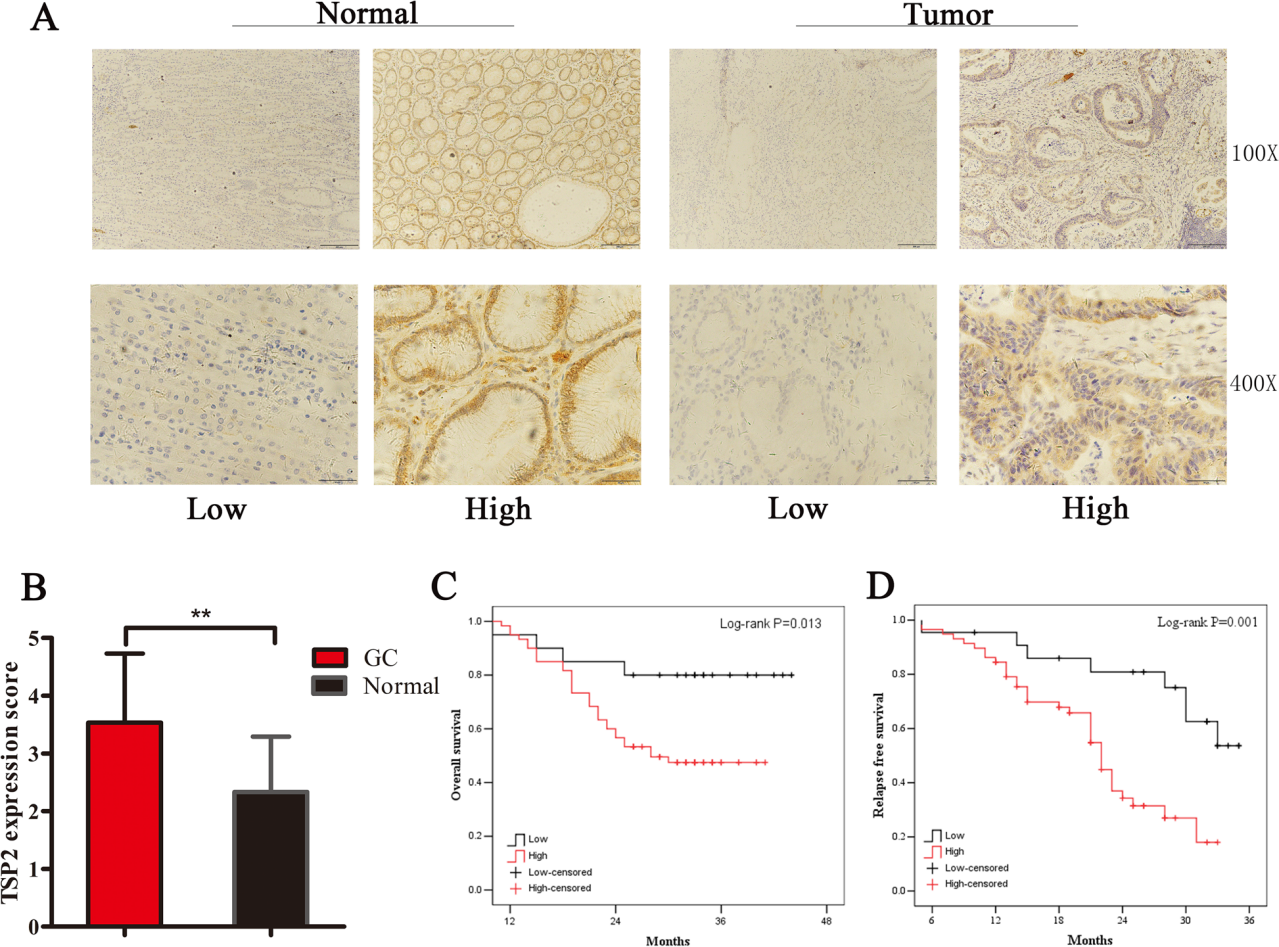
The relationship between TSP2 expression and clinicopathological characteristics in 80 cases of GC tissues: **A** TSP2 immunohistochemical staining of 80 cases of GC tissues showed low expression and high expression. **B** TSP2 expression level in 80 cases of GC tissues (**P<0.01); **C** The effect of TSP2 expression on OS in 80 cases of GC (*P*=0.013) **D** The effect of TSP2 expression on RFS in 80 cases of GC (*P*=0.001)

Kaplan–Meier Plotter database analysis results show that high TSP2 expression significantly affects the overall survival (OS) of GC patients (HR = 1.55, 95% CI: 1.29–1.85; *P <* 0.01) (Supplementary Fig. 2A). Post progression survival (PPS) (HR = 1.51, 95% CI: 1.19–1.9; *P <* 0.01) (Supplementary Fig. 2B) and first progression survival (FP) (HR = 1.53, 95% CI: 1.25–1.88; *P <* 0.01) are also significantly affected by high TSP2 expression (Supplementary Fig. 2C). In addition, Kaplan–Meier analysis was performed on the OS and RFS of 80 GC patients, and statistically significant differences were noted between the TSP2 low expression group and the high expression group (Fig. 1C,D).

### The effect of TSP2 expression on the prognosis of GC patients in different subgroups

Kaplan–Meier Plotter was used to analyse the effect of TSP2 expression on different subgroups of GC patients, and the results showed that the TSP2 expression level affected the OS of patients differently based on sexes, treatment methods, HER2 expression, M staging, Lauren classification, and differentiation type subgroups (*P <* 0.05). TSP2 expression did not affect the prognosis of GC patients in the N0 subgroup (HR = 1.67, 95% CI: 0.73–3.83, *P* = 0.22) but significantly affected the prognosis of GC patients in the N(1 + 2 + 3) subgroup (HR = 2.36, 95% CI: 1.81–3.09, *P <* 0.01). Furthermore, TSP2 did not affect the prognosis of patients with stage I and II GC (*P >* 0.05) but significantly affected the prognosis of patients with stage III and IV GC (*P <* 0.01), as shown in Table 1.

**Table 1:** The effect of TSP2 in the Kaplan-Meier Plotter database on the prognosis of patients with different subgroups of GC

Clinical characteristics	Items	Cases	HR (95% CI)	*P* value
Gender	Female	236	2.05 (1.44–2.92)	4.9e−05
	Male	545	1.52 (1.22–1.89)	0.00015
Treatment	Surgery alone	380	1.71 (1.28–2.29)	0.00023
	5 FU based	153	0.65 (0.46–0.92)	0.014
	Other adjuvant	76	2.82(1.17–6.79)	0.015
HER2 status	Negative	532	1.58(1.25–1.99)	9.4e−05
	Positive	344	1.58 (1.22–2.05)	0.00049
Stage	StageI	67	0.45 (0.17–1.24)	0.11
	StageII	140	0.62(1.34–1.14)	0.12
	StageIII	305	1.89(1.41–2.53)	1.4e−05
	StageIV	148	1.87(1.27–2.77)	0.0014
Stage T	T2	241	1.84(1.18–2.86)	0.0061
	T3	204	1.87(1.32–2.63)	3.0e−04
	T4	38	1.91(0.82–4.47)	0.13
Stage N	N0	74	1.67(0.73–3.83)	0.22
	N (1 + 2 + 3)	422	2.36(1.81–3.09)	8.2e−11
	N1	225	2.31(1.53–3.48)	3.8e−05
	N2	121	2.92(1.84–4.63)	1.9e-6
	N3	76	2.27(1.31–3.91)	0.0026
Stage M	M0	444	2.14(1.62–2.83)	3.9e-8
	M1	56	1.84(1.01–3.33)	0.042
Lauren classification	Instestinal	320	2.49 (1.8–3.46)	1.6e-8
	Diffuse	241	1.89 (1.34–2.66)	0.00023
Differentiation	Mixed	33	4.55 (1.56–13.32)	0.0025
	Poorly	165	1.79 (1.16–2.75)	0.0074
	Moderately	67	2.71 (1.35–5.44)	0.0036
	Well	32	4.36 (1.8–10.54)	4.0e-04

### The relationship between TSP2 expression and clinicopathological characteristics of GC patients

To explore whether TSP2 expression is related to clinicopathological characteristics, immunohistochemistry was used to detect TSP2 expression levels in GC and paracancerous tissues. The representative diagram is shown in Fig. 1A. High TSP2 expression was noted in 76.3% (58/80) of GC tissues. High TSP2 expression was noted in 23.7% (19/80) of normal gastric tissue samples, and the expression level of TSP2 in GC tissue was significantly higher than that in normal tissue adjacent to cancer (Table 2, Fig. 1B). In addition, TSP2 expression levels in GC were significantly positively correlated with TNM staging (*P <* 0.01), lymph node metastasis N staging (*P* = 0.038), and distant organ metastasis pM staging (*P* = 0.025) and positively correlated with the pMMR/MSI-L/MSS ratio (Table 3). In addition, univariate and multivariate analyses showed that TSP2 upregulation is an independent prognostic indicator of OS in patients with gastric cancer (Table 4). These findings indicate that TSP2 has important clinical value in patients with gastric cancer.

**Table 2:** TSP2 expression in 80 cases of gastric cancer tissues (n%)

Tissue	Cases	TSP2 expression		χ^2^	*P*-value
		Low	High		
Normal	80	61(76.3)	19(23.7)	38.079	<0.01
Tumor	80	22(27.5)	58(72.5)

**Table 3:** Relationship between TSP2 expression and clinicopathological characteristics in 80 cases of gastric cancer (n%)

Clinical	Cases	TSP2 expression		χ^2^	*P*-value
characteristics		Low	High		
Age (years)				1.802	0.180
<65	34	12(35.3)	22(64.7)		
≥65	46	10(21.7)	36(78.3)		
Gender				1.863	0.172
Male	41	14(34.1)	27(65.9)		
Female	39	8 (20.5)	31(79.5)		
TNM stage				12.406	<0.01
I/II	33	16(48.5)	17(51.5)		
III/IV	47	6 (12.8)	41(87.2)		
T stage				2.190	0.139
T1/T2	23	9 (39.1)	14(60.9)		
T3/T4	57	13(22.8)	44(77.2)		
N stage				4.296	0.038
N0	33	5 (15.2)	28(84.8)		
N(1 + 2 + 3)	47	17(36.2)	30(63.8)		
M stage				5.035	0.025
M0	68	15(22.1)	53(77.9)		
M1	12	7 (58.3)	5 (41.7)		
Differentiation				0.348	0.555
Poor/undifferentiated	43	13(30.2)	30(69.8)		
Well/moderate	37	9 (24.3)	28(75.7)		
MSI/MMR				16.425	<0.01
pMMR/ MSI-L/MSS	66	12(18.2)	54(81.8)		
dMMR/MSI-H	14	10(71.4)	4 (28.6)		

*Abbreviations*: *pMMR* proficient mismatch repair, *MSI-L* microsatellite instability low, *MSS* microsatellite stable, *dMMR* deficient mismatch repair, *MSI-H* microsatellite instability high

**Table 4:** Univariate and multivariate analyses of overall survival in gastric cancer

Variable	Univariate analysis			Multivariate analysis		
	HR	95% CI	*P* value	HR	95% CI	*P* value
Age	1.011	0.985–1.037	0.423	–	–	–
Gender	0.860	0.438–1.690	0.662	–	–	–
TNM stage	4.132	1.704–10.016	0.002	3.491	1.377–8.848	0.008
T stage	1.838	0.400–1.755	0.639	–	–	–
N stage	1.824	0.418–1.621	0.574	–	–	–
M stage	1.502	0.153–1.643	0.255	–	–	–
Differentiation	1.941	0.478–1.852	0.860	–	–	–
MSI/MMR	0.538	0.189–1.528	0.244	–	–	–
TSP2	3.436	1.208–9.771	0.021	3.243	1.269–8.288	0.014

*HR* hazard ratio, *CI* confidence interval

*p* < 0.05 represents the *p*-values with significant differences

### Effect of TSP2 on GC cell invasion and metastasis in vitro

**Fig. 2:**
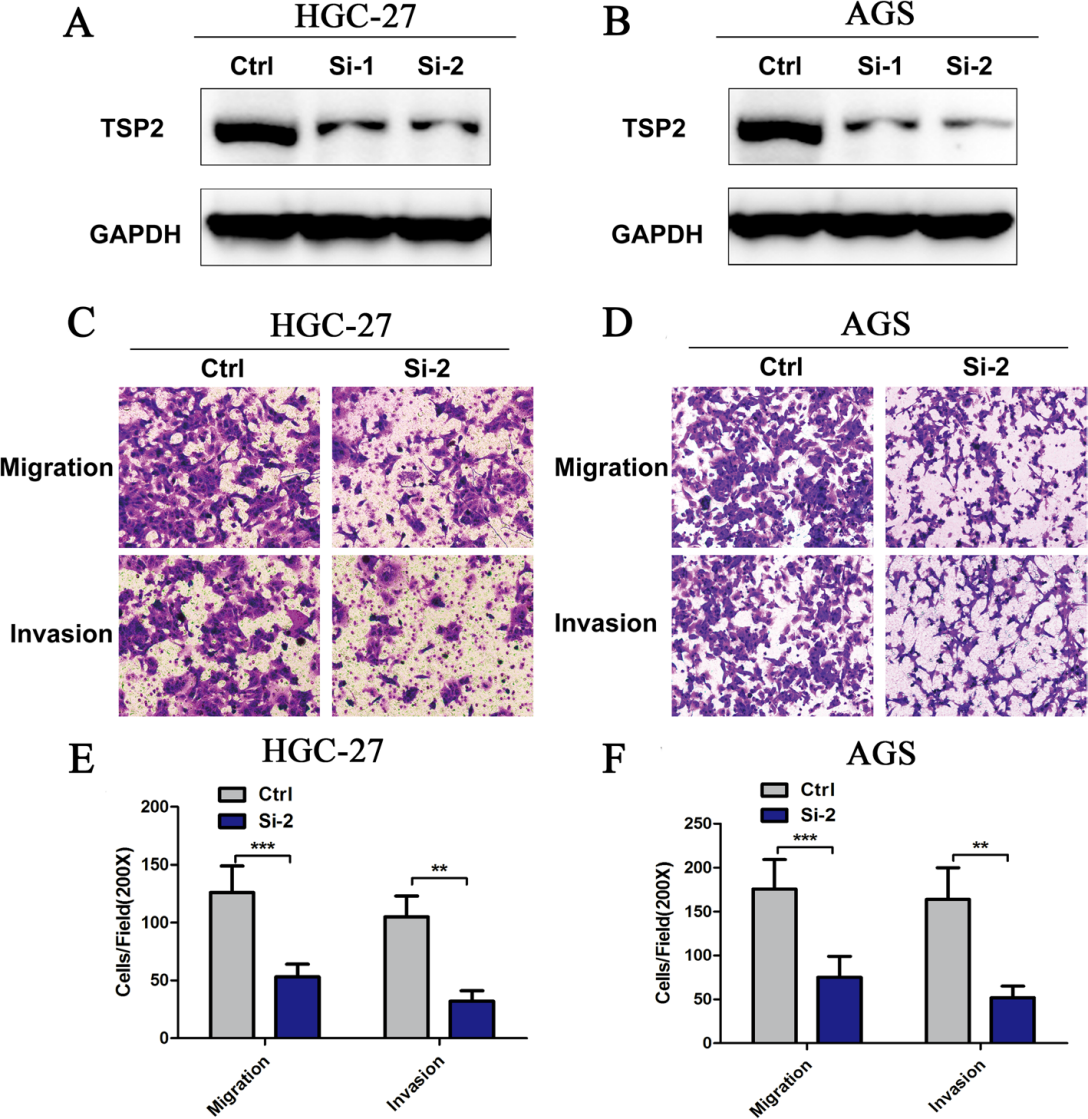
Western blot detection of TSP2 protein expression level: (**A**) HGC27 GC cell line, (**B**) AGS GC cell line, Ctrl: control group; Si-1, Si-2: cell line after transfection. Transwell cell test detects the migration and invasion ability of GC cells: Migration: migration test; Invasion: invasion test; comparison between Ctrl control group and Si-2 experimental group (**P < 0.05; ***P < 0.01)

Western blot results showed that the TSP2 protein levels of HGC27 and AGS cells decreased significantly (Fig. 2A, B). The results of Transwell migration experiments showed that after knocking down the TSP2 expression levels of HGC27 and AGS cells, the invasion and migration ability of cells decreased significantly (Fig. 2C-F). The above results indicate that targeted knockdown of TSP2 can inhibit HGC27 and AGS cell migration and invasion.

### Proliferation and apoptosis in cells transfected with different silencing sequences

**Fig. 3:**
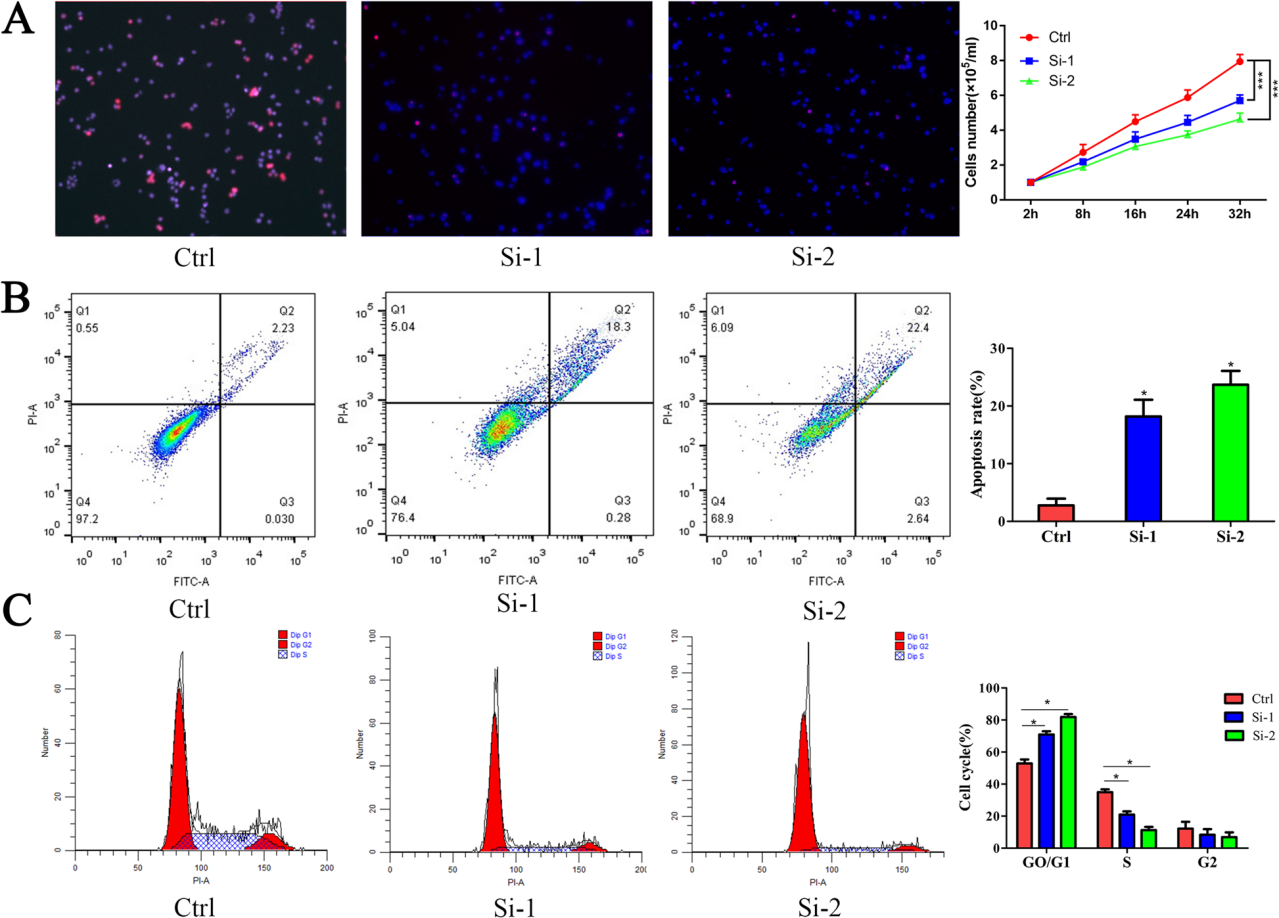
**A**. EdU assay was used to detect the cell proliferation phenotype with the treatment of ethanol or metapristone. **B**. Apoptosis of cells transfected with different silencing sequences measured by flow cytometry. **C**. Cell cycle distribution of cells transfected with different silencing sequences evaluated by flow cytometry

**Fig. 4:**
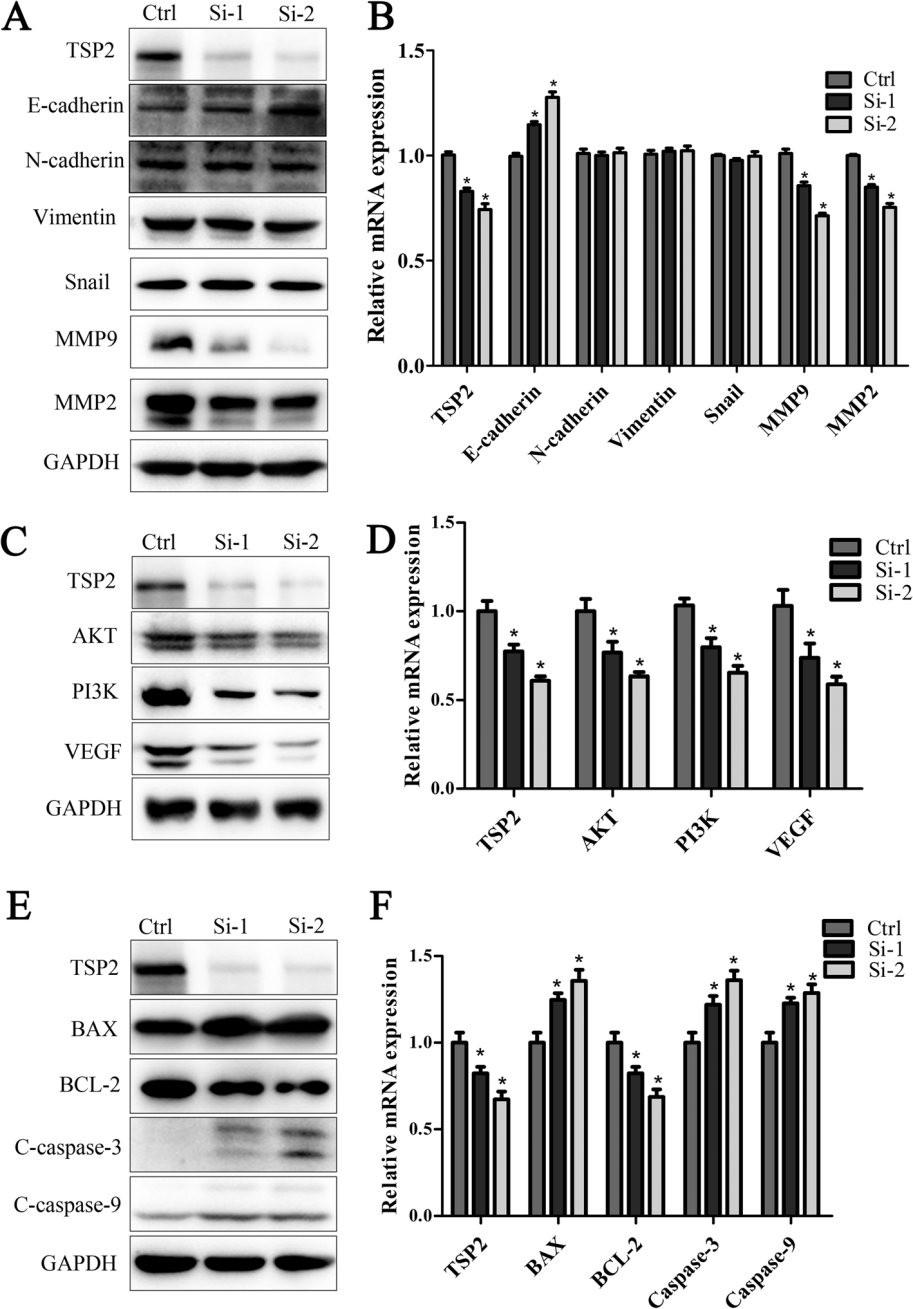
**A**. Knockdown of TSP2 could down-regulate MMPs and up-regulate E-cadherin. **B**. The mRNA expression of migration-related proteins MMP2, MMP9, E-cadherin, N-cadherin, vimentin, and snail. **C**. Knockdown of TSP2 inhibited the expression of AKT, PI3K and VEGF. **D**. The mRNA expression of AKT, PI3K and VEGF. **E**. The expression of apoptosis proteins cleaved caspase-3, cleaved caspase-9, Bcl-2 and Bax. **F**. The mRNA expression of apoptosis proteins caspase-3, caspase-9, Bcl-2 and Bax

First, EdU experiment results demonstrate that TSP2 promotes HGC27 cell proliferation. The cell growth curve results showed that the growth and proliferation of the si-1 and si-2 cell lines after knockdown was significantly slower than that of the control group (Fig. 3A). Second, flow cytometry results showed that compared with the control group, the two knockdown cell lines Si-1 and Si-2 had more cells in G0/G1 phase and fewer cells in S phase (*P* < 0.05, Fig. 3B). These results indicate that TSP2 silencing inhibits the cell cycle of gastric cancer cells, and the cells are stagnant in the G0/G1 phase, indicating that the proliferation ability is inhibited. Compared with the control group, the apoptosis rate of the si-1 and si-2 groups was significantly increased (*P* < 0.05, Fig. 3C). These results indicate that silencing of TSP2 expression significantly promotes gastric cancer cell apoptosis. Wb technology was used to detect the expression of apoptosis proteins cleaved caspase-3, cleaved caspase-9, Bcl-2 and Bax. The results showed that after knocking down the expression of TSP2, the expression of Bcl-2 protein was significantly down-regulated and the expression of cleaved caspase-3, cleaved caspase-9 and Bax was significantly up-regulated (Fig. 4E). RT-qPCR technology was used to detect the mRNA expression of apoptosis proteins caspase-3, caspase-9, Bcl-2 and Bax. The results showed that after knocking down the expression of TSP2, the mRNA expression of Bcl-2 protein was significantly down-regulated and the mRNA expression of caspase-3, caspase-9 was significantly up-regulated (Fig. 4F).

### TSP2 knockdown downregulates the expression of HGC27 cell migration-related proteins

Wb technology was used to detect the expression of the following migration-related proteins: MMP2, MMP9, E-cadherin, N-cadherin, vimentin, and snail. After knocking down TSP2 expression, the expression of E-cadherin, N-cadherin, vimentin, and snail did not change significantly, whereas MMP2 and MMP9 protein expression was significantly downregulated and the expression of E-Cadherin was significantly up-regulated (Fig. 4A). RT-qPCR technology was used to detect the mRNA expression of migration-related proteins MMP2, MMP9, E-cadherin, N-cadherin, vimentin, and snail. The results showed that after knocking down the expression of TSP2, the mRNA expression of MMP2, MMP9 protein was significantly down-regulated and the mRNA expression of E-cadherin was significantly up-regulated (Fig. 4B).

### TSP2 promotes GC cell mobility through the VEGF/PI3K/AKT signalling transduction pathway

The AKT signalling pathway plays a very important role in cell proliferation and migration. After knocking down TSP2 expression, the expression of the related proteins AKT, PI3K and VEGF in the AKT signalling pathway was detected by WB. The results showed that knocking down TSP2 significantly downregulated the expression of AKT, PI3K and VEGF (Fig. 4C). The above experimental results indicate that TSP2 may regulate the biological behaviour of HGC27 by regulating the AKT signalling pathway.

RT-qPCR technology was used to detect the mRNA expression of signalling pathway proteins AKT, PI3K and VEGF. The results showed that after knocking down the expression of TSP2, the mRNA expression of AKT, PI3K and VEGF protein was significantly down-regulated (Fig. 4D).

### Correlation between TSP2 gene expression and mismatch repair gene expression in GC

GEPIA database analysis results showed that the expression level of TSP2 in gastric adenocarcinoma (stomach adenocarcinoma; STAD) was significantly positively correlated with the expression of a series of mismatch repair genes, PMS2, MSH6, MSH2, and MLH1 (*P* < 0.05, Supplementary Fig. 3).

## Discussion

GC is one of the common types of gastrointestinal tumours. Its occurrence is related to various factors, including genetic factors and nongenetic factors, and genetic factors play an essential role in the pathogenesis of GC [2]. The specific mechanisms involved in the pathogenesis of GC are worthy of further investigation. Although the comprehensive treatment of GC has made meaningful progress, more accurate prognostic markers are lacking. This study used bioinformatics technology combined with clinical experiments to determine that TSP2 expression is significantly increased in GC. Simultaneously, the high expression of TSP2 significantly affects the prognosis of GC patients and significantly affects the prognosis of GC patients with lymph node metastasis. This study also determined that TSP2 expression levels are related to a variety of genes that affect the biological behaviour of GC, which provides a theoretical direction and data support for the further study of TSP2 in GC.

The occurrence of tumours is the result of multiple factors. Tumourigenesis is a multi-stage process that allows tumour cells have unlimited self-proliferation ability and the ability to escape apoptosis [23]. Tumourigenesis also requires continuous nutritional support, including blood vessel growth and infiltration [24, 25]. New blood vessels are vital for tumour cell growth, infiltration, and metastasis [5]. When the normal balance between inhibiting tumour angiogenesis and promoting tumour angiogenesis is broken, numerous tumour blood vessels are rapidly formed, and tumour cells gain the ability for unlimited proliferation, infiltration, and metastasis. However, the blood vessels of the tumour are new and abnormal [26]. TSP2 is a member of the TSP family. It is a new, naturally occurring inhibitor of angiogenesis [27]. TSP significantly inhibits the expression of vascular endothelial growth factor (VEGF) [28]. It is currently the most studied member of the TSP family, has the most complex functions and is closely related to various cell behaviours. Related studies have shown that TSP2 is synthesized, secreted, and transported to the corresponding extracellular matrix by fibroblasts, smooth muscle cells, endothelial cells, keratinocytes, and other types of cells [29]. Given that each subunit of TSP2 is composed of many discrete domains, these different domains can mediate TSP2 binding to various ligands, such as heparin, collagen, fibronectin, plasminogen, and plasminogen activation [30]. Related studies have confirmed that at least two different receptors interact with these ligands. The role of receptors and ligands is precisely mediated by the above domains. TSP2 participates in a variety of cell behaviours, including cell adhesion, migration, and proliferation [31]. Therefore, TSP2 is considered to play an essential role in cell growth and embryonic development and angiogenesis.

In this study, GEPIA and Kaplan–Meier Plotter database systems were used to analyse TSP2 expression levels in GC to understand the relationship between their expression and clinical prognosis. The results showed that TSP2 expression in GC tissues was significantly higher than that in normal tissues. TSP2 expression mainly affects the clinical prognosis of GC patients with lymphatic stages N1–3. This finding indicates that high TSP2 expression is closely related to lymph node metastasis and tumour invasion in GC. In addition, high TSP2 expression affects the clinical prognosis of GC patients in various GC subgroups, indicating that TSP2 represent a potential biomarker for predicting tumour prognosis in GC. To further clarify the expression of TSP2 in GC tissues, this study performed immunohistochemical analysis on 80 pairs of fresh GC tissues and adjacent tissues. The results showed that the TSP2 expression level was significantly related to TNM staging (*P* < 0.01), pM staging of distant metastasis (*P* = 0.025), and other clinicopathological characteristics but not to sex or age (*P* > 0.05). Kaplan–Meier survival analysis results showed that patients with high TSP2 expression had a shorter survival time than patients with low expression, were more likely to relapse, and had a worse prognosis. In addition, univariate and multivariate analyses showed that TSP2 upregulation is an independent prognostic indicator of OS in patients with gastric cancer. Elevated TSP2 expression is an independent risk factor for poor prognosis in patients. These findings indicate that TSP2 has important clinical value in patients with gastric cancer. Given that the cohort of 80 pairs of GC tissues includes only twelve cases of M1 disease, such a low-metastasis disease group may lead to limited statistical results. Considering that our research was conducted in a small group of patients, it is necessary to conduct larger-scale prospective clinical studies to completely understand and develop the prognostic and therapeutic value of TSP2.

To explore the role of TSP2 in the metastatic activity of GC cells, this study selected the GC cell lines HGC27 and AGS for transfection. The results showed that the expression of Si-1 and Si-2 histones was significantly lower than that of the control group, verifying the success of the transfection model setup. Transwell cell migration and invasion experiments revealed that TSP2 gene expression was reduced after interfering with the TSP2 gene in GC cells, and the cell invasion and metastasis ability was reduced. The migration ability of tumour cells is mainly regulated by the matrix metalloproteinase (MMP) protein family. Members of the MMP family degrade the extracellular matrix and participate in signal transduction. This family is mainly involved in cell migration and invasion, and expression of MMP proteins is significantly upregulated in a variety of tumour cells. This study also found that after altering TSP2 expression, MMP9 protein expression was significantly downregulated. Furthermore, the lack of expression of cadherin will lead to a decrease in the adhesion ability of tumor cells, and then metastasis and spread to surrounding tissues.

TSP2 silencing results in the inhibition of the proliferation of gastric cancer cells and cell migration. In addition, cells transfected with the silencing sequence undergo apoptosis. The AKT signalling pathway is involved in a variety of physiological activities in cells. The AKT signalling pathway regulates cell proliferation by regulating the expression of cyclin and cyclin-dependent kinases. The AKT signalling pathway also inhibits cell apoptosis by inhibiting the expression of P53. In addition, the PI3K/AKT signalling pathway also regulate the migration ability of cells by regulating the expression of MMP proteins. In addition, the development of a new blood vessel supply or angiogenesis plays a vital homeostasis role given that blood vessels transport nutrients to tissues and organs and remove catabolic products. However, uncontrolled blood vessel growth can promote many disease processes, including tumourigenesis. The discovery of vascular endothelial-derived growth factor (VEGF) has completely changed our understanding of angiogenesis during development and physiological homeostasis, and VEGF mainly targets endothelial cells [32]. Further studies have found that the downregulation of TSP2 leads to downregulation of AKT signalling pathway-related proteins AKT/PI3K/VEGF, thereby regulating tumour angiogenesis, tumour cell proliferation and migration and many other physiological activities. However, the specific mechanisms still need to be further explored. In conclusion, this experiment shows that TSP2 gene expression promotes GC cell proliferation, migration and invasion.

On the other hand, chromosomal instability and microsatellite instability are two key mechanisms involved in the occurrence of gastrointestinal tumours [33, 34]. The mismatch repair system (MMR) is responsible for maintaining genome stability. When MMR function is abnormal, the weakened or even missing function of the MMR system will cause microsatellite instability [35]. Microsatellites change, and the total mutation rate of specific cells increases [36]. Therefore, MMR plays a vital role tumourigenesis and the biological behaviour of tumours [37]. The current use of immune checkpoint inhibitor therapy for tumours is referred to as the “MSI era” because microsatellite instability (MSI) or mismatch repair gene status (MMR) is currently the best predictor of efficacy [38, 39]. Based on the MSI status, GC patients can be divided into two groups according to the efficacy of immunotherapy: “dominant population” MSI-H/dMMR GC cancer (MSI-H) and “ineffective population” MSS/pMMR GC cancer (MSS) [40]. Mismatch repair gene family members include *MLH1, MSH2, MSH6,* and *PMS2* [41], all of which play an essential role in GC occurrence and progression [42]. Through gene correlation analysis, this study also found that the TSP2 gene has a significant correlation with the expression of four crucial genes in the mismatch repair family, further confirming that the TSP2 gene has potential guiding significance for clinical molecular typing and treatment options in GC.

## Conclusion

In summary, this study analysed the correlation between TSP2 and GC through bioinformatics technology combined with clinical samples and in vitro experiments. TSP2 was highly expressed in GC and had a cancer-promoting effect. Downregulating its expression by exogenous means results in the inhibition of GC proliferation, migration, and invasion. High TSP2 expression affects the prognosis of GC patients, and TSP2 expression is closely related to the poor prognosis of patients with GC lymph node metastasis. TSP2 is a potential marker for GC lymph node metastasis and patient prognosis.

## Supplementary Information

**Additional file 1: Supplementary Figure 1**. Analysis expression level of TSP-2 in different tumors and GC. Analyze the results with the GEPIA database: (A) Dot plots of gene expression profiles in all tumor samples and matched normal tissues; (B) Comparison of expression levels of TSP2 gene in GC and normal stomach tissues (*P* < 0.01); (C) The gene expression profile bar graphs of all tumor samples and paired normal tissues. The height of the bar represents the median expression of certain tumor types or normal tissues, where Stomach adenocarcinoma (STAD,T = 22.67; *N* = 1.45); (D) The expression of TSP2 gene in GC clinical stage (*P* < 0.01). **Supplementary Figure 2.** Kaplan-Meier Plotter database analysis of the relationship between TSP2 mRNA expression level and prognosis in GC patients: (A) TSP2 expression affects OS of GC patients, (B) Progressive survival after recurrence (PPS) and (C) First progression survival (FP). **Supplementary Figure 3.** The correlation between TSP2 gene and mismatch repair gene expression in the GEPIA database. (A-D) respectively indicate a significant positive correlation with the mismatch repair genes *PMS2, MSH6, MSH2, MLH1*, and TSP2 (THBS2) genes. **Supplementary Figure 4**. A. Original figure about Western-blot results showed that the TSP2 protein levels of HGC27 and AGS cells. The samples derive from the same experiment and that gels/blots were processed in parallel. B, C and D, Original figure about Western-blot results showed that the TSP2 protein levels of HGC27 cells. The samples derive from the same experiment and that gels/blots were processed in parallel.

## Data Availability

All analyzed data are included in this published article and its supplementary information file. The data underlying this study are freely available from GEPIA data portal (http://gepia.cancer-pku.cn/index.html) and the Kaplan-Meier Plotter prognostic analysis tool (http://kmplot.com/analysis/). The authors did not have special access privileges. The original data are available upon reasonable request to the corresponding author.

## Abbreviations

TSP2: THBS2,Thrombospondin 2
TSP: Thrombospondin
GC: Gastric Cancer
STAD: Stomach adenocarcinoma
GEPIA: Gene Expression Profile Interactive Analysis
UICC: The International Union Against Cancer
NCCN: The National Comprehensive Cancer Network
OS: Overall survival
RFS: Relapse free survival
PPS: Post progression survival
FP: First progression survival
VEGF: Vascular endothelial growth factor
pMMR: Proficient mismatch repair
MSI-L: Microsatellite instability low
MSS: Microsatellite stable
dMMR: Deficient mismatch repair
MSI-H: Microsatellite instability high
