# Supplementary material for: Thrombospondin-2 holds prognostic value and is associated with metastasis and the mismatch repair process in gastric cancer
Source: BMC Cancer. 2022 Mar 7;22:250. doi: 10.1186/s12885-022-09201-3 (PMC8900425; doi:10.1186/s12885-022-09201-3)

Thrombospondin-2 Holds Prognostic Value and is Associated with Metastasis and the Mismatch Repair Process in Gastric Cancer

Xiao-dong Chu[1a], Zheng-bin Lin[1a],Ting Huang[2], Hui Ding[1],Yi-ran Zhang[1], Zhan Zhao[1], Shu-chen Huangfu[1], Sheng-hui Qiu[1], Yan-guan Guo[1], Xiao-li Chu[3], Jing-hua Pan[1*], Yun-long Pan[1*].

[1]Department of General Surgery, the First Affiliated Hospital of Jinan University, Guangzhou, 510632, China.

[2]Department of Clinical Pathology, First Affiliated Hospital of Jinan University, Guangzhou, 510630, China.

[3]Guangdong Provincial Key laboratory of Chinese Medicine for Prevention and Treatment of Refractory Chronic Diseases, The Second Affiliated Hospital of Guangzhou University of Chinese Medicine, Guangzhou, 510120, China

[a]Author contributed equally

Disclosures of potential conflicts of interest may be found at the end of this article.

*Corresponding author:

Prof. Jing-hua Pan, PhD

Department of General Surgery, the First Affiliated Hospital of Jinan University,

613 Huangpu West Avenue, Guangzhou, Guangdong, China.

Tel: 86-20-38688609; E-mail: huajanve@foxmail.com.

Supplementary Figure 1. Analysis expression level of TSP-2 in different tumors and GC. Analyze the results with the GEPIA database: (A) Dot plots of gene expression profiles in all tumor samples and matched normal tissues; (B) Comparison of expression levels of TSP2 gene in GC and normal stomach tissues (*P*<0.01); (C) The gene expression profile bar graphs of all tumor samples and paired normal tissues. The height of the bar represents the median expression of certain tumor types or normal tissues, where Stomach adenocarcinoma (STAD ,T=22.67; N=1.45); (D) The expression of TSP2 gene in GC clinical stage (*P*<0.01).

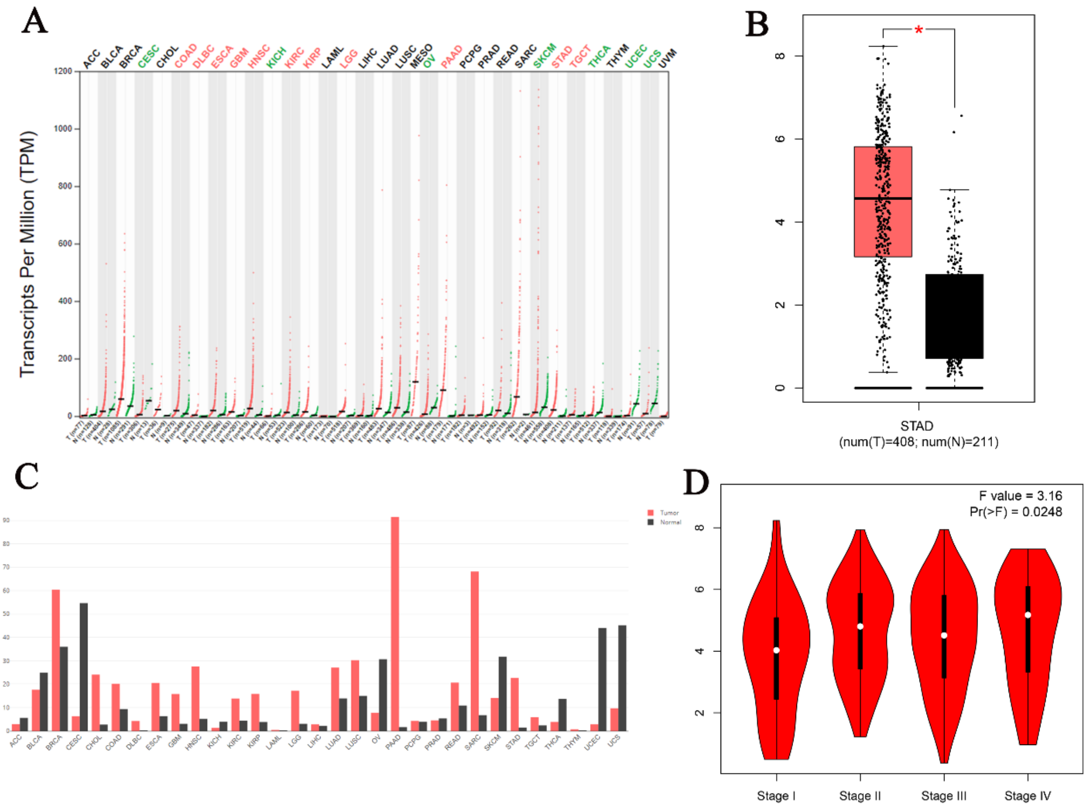

Supplementary Figure 2. Kaplan-Meier Plotter database analysis of the relationship between TSP2 mRNA expression level and prognosis in GC patients: (A) TSP2 expression affects OS of GC patients, (B) Progressive survival after recurrence (PPS) and (C) First progression survival (FP).

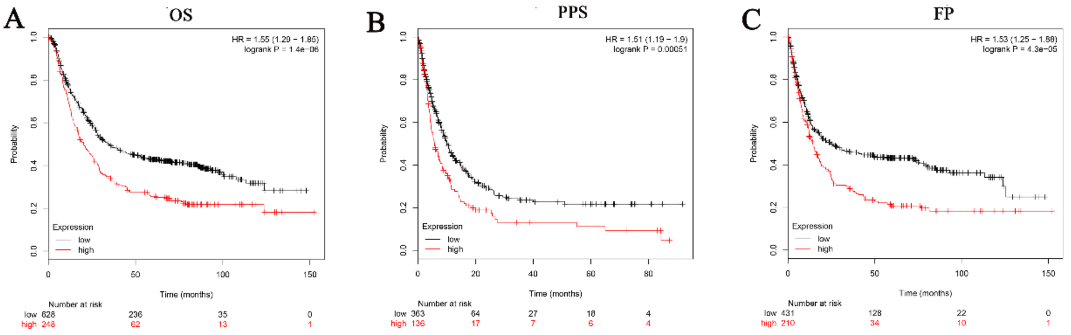

Supplementary Figure 3. The correlation between TSP2 gene and mismatch repair gene expression in the GEPIA database. (A-D) respectively indicate a significant positive correlation with the mismatch repair genes *PMS2, MSH6, MSH2, MLH1*, and TSP2 (THBS2) genes.

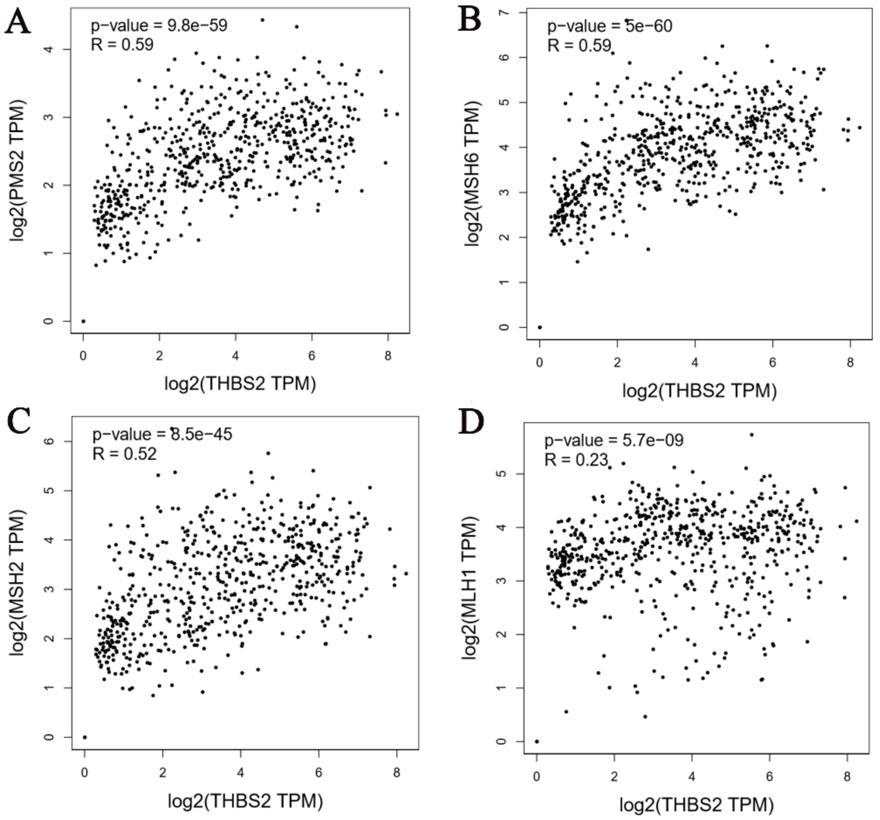

Supplementary Figure4. A. Original figure about Western-blot results showed that the TSP2 protein levels of HGC27 and AGS cells. The samples derive from the same experiment and that gels/blots were processed in parallel. B, C and D, Original figure about Western-blot results showed that the TSP2 protein levels of HGC27 cells. The samples derive from the same experiment and that gels/blots were processed in parallel.

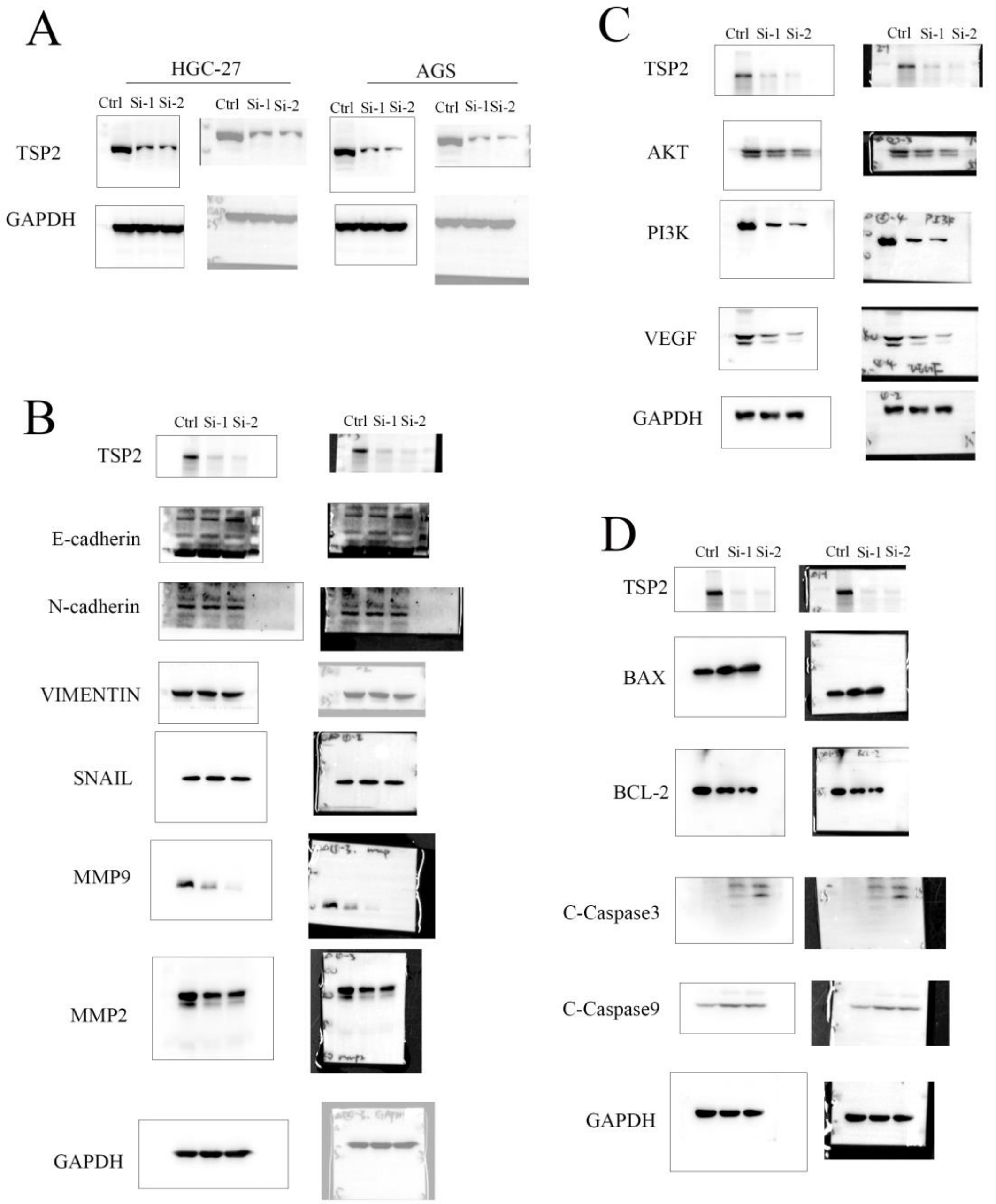

Supplement: Supplementary file 1 — Additional file 1: Supplementary Figure 1. Analysis expression level of TSP-2 in different tumors and GC. Analyze the results with the GEPIA database: (A) Dot plots of gene expression profiles in all tumor samples and matched normal tissues; (B) Comparison of expression levels of TSP2 gene in GC and normal stomach tissues (P < 0.01); (C) The gene expression profile bar graphs of all tumor samples and paired normal tissues. The height of the bar represents the median expression of certain tumor types or normal tissues, where Stomach adenocarcinoma (STAD,T = 22.67; N = 1.45); (D) The expression of TSP2 gene in GC clinical stage (P < 0.01). Supplementary Figure 2. Kaplan-Meier Plotter database analysis of the relationship between TSP2 mRNA expression level and prognosis in GC patients: (A) TSP2 expression affects OS of GC patients, (B) Progressive survival after recurrence (PPS) and (C) First progression survival (FP). Supplementary Figure 3. The correlation between TSP2 gene and mismatch repair gene expression in the GEPIA database. (A-D) respectively indicate a significant positive correlation with the mismatch repair genes PMS2, MSH6, MSH2, MLH1, and TSP2 (THBS2) genes. Supplementary Figure 4. A. Original figure about Western-blot results showed that the TSP2 protein levels of HGC27 and AGS cells. The samples derive from the same experiment and that gels/blots were processed in parallel. B, C and D, Original figure about Western-blot results showed that the TSP2 protein levels of HGC27 cells. The samples derive from the same experiment and that gels/blots were processed in parallel. [file 12885_2022_9201_MOESM1_ESM.pdf]
